# Insecticide Resistance and Fitness: The Case of Four* Aedes aegypti* Populations from Different Brazilian Regions

**DOI:** 10.1155/2018/6257860

**Published:** 2018-10-09

**Authors:** Mariana Rocha David, Gabriela Azambuja Garcia, Denise Valle, Rafael Maciel-de-Freitas

**Affiliations:** ^1^Laboratório de Mosquitos Transmissores de Hematozoários, Instituto Oswaldo Cruz, Fiocruz, Brazil; ^2^Laboratório de Biologia Molecular de Flavivírus, Instituto Oswaldo Cruz, Fiocruz, Brazil; ^3^Instituto Nacional de Ciência e Tecnologia em Entomologia Molecular, Brazil

## Abstract

**Background:**

Chemical control is still a major strategy to constrain vector density and mitigate pathogen transmission. However, insecticide overuse poses a high selective pressure, favouring the spread of resistance alleles in natural populations. In an insecticide-free environment, a fitness cost is expected in resistant insects when compared to susceptible counterparts. This study investigates whether insecticide resistance to an organophosphate (temephos) and a pyrethroid (deltamethrin) is associated with fitness traits in four* Aedes aegypti* wild populations sampled every three months over one year.

**Findings:**

We measured development time from larvae to adult, female survival, wing length, fecundity, and adult resistance to starvation in field insecticide resistant* Ae. aegypti *populations four times over a year. These results were confronted with resistance levels to temephos and deltamethrin and with potentially related mechanisms, including a* kdr* mutation in the pyrethroid target site. No differences in fitness cost were found after contrasting mosquitoes from the same population collected throughout a year, irrespective of differences in insecticide resistance levels. Additionally, significant differences were not observed among field populations. However, compared to the reference strain Rockefeller, field females survived significantly less. Moreover, larval development was equal or slower in three out of four field populations. In no case differences were evidenced in starvation tolerance, wing length, and fecundity.

**Conclusions:**

Overall, field resistant mosquitoes seemed to have a slight fitness disadvantage when compared with the Rockefeller susceptible strain which might represent a potential fitness cost of insecticide resistance. However, after comparing* Ae. aegypti *from the same population but sampled at different moments, or from different field populations, mosquito life-history traits varied independently of resistance ratios. The metabolic deviations necessary to overcome the adverse effects of insecticides may cause an energy trade-off that affects energy allocation and ultimately basic demands of insect biology. The extent of fitness cost due to insecticide resistance is critical information to delay the evolution of resistance in wild vector populations.

## 1. Background

Arbovirus transmission has become one of the major global public health issues in the past decades. Dengue virus (DENV) is estimated to affect annually about 390 million people worldwide [1], causing more illness and death than any other virus transmitted by arthropods. In 2013-14, chikungunya virus (CHIKV) was introduced into the Caribbean and Latin America infecting ~450,000 people three years later [2]. Zika virus (ZIKV) is believed to have invaded South America from French Polynesia in the same period [3, 4], causing ~215,000 infections in Brazil [5]. ZIKV is associated with congenital disorders and neurological complications such as Guillain-Barré syndrome, with major neurological and cognitive disabilities [6, 7]. All the evidence so far points to the fact that DENV, CHIKV, and ZIKV are primarily transmitted by* Aedes aegypti*, an essentially urban mosquito [8, 9].

Since there is currently no vaccine available for CHIKV or ZIKV, disease prevention relies exclusively upon actions directed toward the insect vector. Today, control efforts focus essentially on maintaining mosquito populations below a critical threshold to avoid arboviruses outbreaks [10]. Among the vector control approaches available, chemical control is a major strategy in endemic areas through larvicide application in* Aedes *man-made breeding sites and/or vehicle-mounted ultralow volume (ULV) space spraying of adulticides [11–13]. In Brazil, until recently, the organophosphate temephos and the pyrethroid deltamethrin were the major insecticides employed against* Ae. aegypti* larvae and adults, respectively [13]. However, insecticide overuse poses a selective pressure on wild mosquito populations, favouring the frequency of insecticide resistant alleles, gradually impairing chemical control effectiveness. Insecticide resistance alleles confer a great selective advantage whenever chemical control is prioritized, resulting in a sharp increase in resistance levels [14, 15]. Nevertheless, resistance alleles are rarely detected in high frequencies in these insect populations when insecticides are absent from the environment [16]. Therefore, an association between the frequency of resistance alleles and fitness costs is expected [16, 17].

Some hypotheses can explain possible negative fitness effects of resistance, which are as follows: (i) large phenotypic changes can often be deleterious within the context of the previous insecticide-free environment [17]; (ii) the maintenance of metabolic pathways compatible with a resistant status may reallocate energetic resources from other life-history traits, e.g., egg production and/or survival [18]; and (iii) changes in the insecticide-target nervous system proteins may result in fitness disadvantage in insecticide resistant mosquitoes [11, 19, 20]. These phenomena have been observed for agricultural pests and disease vectors [21–24], including* Ae. aegypti* [25–27].

Knowledge of the evolutionary cost of insecticide resistance in* Ae. aegypti *populations is essential to manage the dissemination of such condition. Studies aiming to investigate resistance cost often repeatedly backcross resistant insects with those that are susceptible [11, 28, 29] or artificially select a sample of a field population by exposition to insecticide for several generations [25, 27, 30]. However, it is challenging to estimate the fitness cost of resistant field populations as changes in mosquito life-history traits can be easily influenced and confused by a variety of other genetically determined characteristics beyond those directly related to insecticide resistance [31, 32].

Resistance levels are expected to vary over time according to insecticide selective pressures [14, 15]. In this scenario, samples from the same mosquito populations collected over time could reveal* Ae. aegypti *life-history trait variation in accordance with insecticide resistance fluctuation. Therefore, we investigated how insecticide resistance to an organophosphate (temephos) and a pyrethroid (deltamethrin) is associated with fitness traits in four* Ae. aegypti* wild populations sampled every three months over one year.

## 2. Methods

### 2.1. *Mosquito Populations and Field Collection*

Laboratory tests were conducted with the F1 generation of different Brazilian* Ae. aegypti* populations. Four midsized, Brazilian cities apart from each other by a minimum of 1,180 Km were chosen for mosquito collection to represent different biomes, demography, climatic conditions as well as epidemiological and entomological histories (Table 1). Detailed information about insecticide use during sampling period is described in [33]. For each city, four samplings were performed over one year with an interval of 3 months. For each of the 16 samplings (four localities and four collections), the parental generation was collected with 360 ovitraps baited with hay infusion [34, 35] placed in peridomestic, shaded environments, and arranged in three 1 km^2^ areas per city, located around 10 Km apart from each other. In the laboratory, eggs were reared until the adult stage when specimens were species level identified following the identification key proposed by [36]. At least 500* Ae. aegypti* adult females were enlisted to initiate laboratory colonies from each sampling in order to ensure our sampling procedure was representative of the natural genetic diversity of each population [37]. The Rockefeller strain, laboratory established around 1935, was adopted both as an internal control of all fitness assays and an insecticide susceptible reference lineage [38].

### 2.2. *Insecticide Resistance Levels and Resistance Mechanisms*

The resistance status to the organophosphate temephos and the pyrethroid deltamethrin was reported [33]. Briefly, field L3* Ae. aegypti* larvae (F1) were submitted to quantitative bioassays to evaluate the temephos susceptibility status using eight to ten insecticide concentrations, varying from 0.006 to 0.072 mg/L. Mortality was registered 24 hours after temephos exposure. For each sample three assays with four replicates each (N= 10-20 larvae per replicate) were performed, following WHO recommendations [39]. Quantification of adult resistance to deltamethrin was also performed through dose-response assays using insecticide impregnated papers, according to an adaptation of WHO protocols with ten different deltamethrin concentrations per assay, varying between 2.1 and 109.6 mg/m^2^. Papers were impregnated in the laboratory as previously described [11–13]. Mortality was registered after one hour of exposure and 24 hours of recovery. In each case, three independent assays were performed, with three replicates per concentration, each one containing 15 to 20 adult females [11, 26, 40]. Resistance ratios (RR) were acquired dividing the results obtained for each population by the equivalent Rockefeller's values. In all cases the criterion utilized by the Brazilian MoH to temephos evaluation was employed. According to this criterion, populations with RR_95_ above 3.0 are considered resistant [13, 33].

The activity of enzymes potentially involved in insecticide detoxification (glutathione-S-transferases, GST; esterases, EST; and mixed function oxidases, MFO) were quantified in adult females according to adaptations from WHO and CDC protocols described elsewhere [41, 42]. Three substrates were employed for EST: *α*- and *β*-naphthyl and *ρ*-nitrophenyl acetates, accounting, respectively, for activities named *α*-EST, *β*-EST, and *ρ*NPA-EST. In order to evaluate alterations in the detoxification pathway, 80 to 120 non-blood-fed young females (up to 24 hours after emergence), stored at -80°C, were individually analysed. According to criteria previously defined, the 99th^th^ percentile of the susceptible control strain Rockefeller (p99Rock) was calculated for the activity of each enzyme class; field population data were classified as follows: enzyme activity of any given population was considered unaltered when 0–15% specimens remained beyond p99Rock; values between 15 and 50% and above 50% were classified as altered or highly altered, respectively [41]. Allele-specific PCR was conducted to investigate the presence of the Val1016Ile* kdr* mutation in the pyrethroid target site, the voltage gated sodium channel (Na_V_), in adult males from the parental generation [43, 44]. Females were not recruited in order to avoid the risk of potential contamination with DNA from sperm in the spermathecae.

### 2.3. Fitness Assays

Assays were conducted on four occasions for the mosquitoes collected in each city with the same samples submitted to the insecticide resistance bioassays. Rockefeller mosquitoes were always evaluated simultaneously. In all cases, specimens were kept in incubators at 27.6 ± 0.6°C and 70±10% RH, with both parameters verified twice a day.

#### 2.3.1. Immature Development Time, Starvation Tolerance, and Wing Length

The immature development time refers to the period, in hours, elapsed from larvae to adult. We opted to individually rear mosquitoes to avoid any effects of competition for food [45]. For each sampling, we reared 120 F1 field-derived and 36 Rockefeller specimens, with the larvae monitored three times a day (08:00, 12:00, and 17:00). The assays were conducted in 12-well tissue culture plates. Each well was filled with 4 ml of dechlorinated water receiving 100 *μ*L of a dry yeast suspension daily (Prolev, Recife, PE), containing 0.04 mg on days 01 and 02, 0.08 mg on day 03, 0.16 mg on day 04, 0.32 mg on day 05, 0.64 mg on day 06, and 0.32 mg for the remaining days until the pupae stage [46]. The pupae were then individually transferred to cylindrical plastic tubes (6.5 cm height, 2.5 cm diameter) in which they emerged as adults.

All adult mosquitoes received wet moistened cotton swabs without any nutrient to avoid death by dehydration. Starvation tolerance (i.e., survival without any nutrition) was monitored twice a day (08:00 and 17:00) up to mosquito death, when sex and wing length were registered.

Due to the variation in Rockefeller immature development assays despite identical rearing conditions, each field sampling was normalized by its corresponding Rockefeller specimens to enable comparison among different experimental groups. Normalization procedure is detailed in the Supplementary Materials (Supplementary Text 1). Thus, values generated for the experimental groups, called “scaled development time”, are a measure of how much each field population development time deviates from its corresponding Rockefeller referential. Values above and below 1.0 indicate, respectively, slower and faster development than the Rockefeller strain. Statistics were calculated on these scaled time values which we refer to as “scaled developmental time”.

#### 2.3.2. Female Survival and Fecundity

Pools of 100 larvae were reared in plastic basins filled with 1 L of dechlorinated water. Larvae received 500 mg of dry yeast (Prolev, Recife, PE, Brazil) on day 1 and 250 mg on day 4, after which pupae were transferred to cardboard cages. Following 3-4 days after adult emergence, one-cohort of 60 females from each sampling and 10 Rockefeller females were randomly selected and individually transferred to labeled cylindrical plastic vials (6.5 cm height, 2.5 cm diameter), covered by mosquito netting. A moistened cotton swab overlaid with filter paper on the bottom of the vials served as substrate for oviposition. Mosquitoes received 10% sugar solution* ad libitum*, except during each 24-hour period prior to the blood meals weekly offered from an anesthetized mouse. Three to four days after blood-feeding, filter papers were replaced and the number of eggs was recorded. Mosquito survival was scored daily until the 60th day.

### 2.4. Statistical Analysis

The biological parameters were compared with insecticide resistance levels (resistance ratios 95, or RR_95_, i.e., the ratio between the insecticide lethal concentrations that kill 95% of tested mosquitoes and the Rockefeller strain) in order to investigate potential fitness costs of insecticide resistance in the* Ae. aegypti *mosquitoes. Wing length and starvation tolerance were analysed using female data. Fecundity was the number of eggs laid in the first week of monitoring considering females that laid at least one egg. Associations among these biological parameters and the* kdr* Val1016Ile substitution in the pyrethroid target site, the Na_V_ gene, were performed (Supplementary Figure 1); associations with the activity of enzymes related to metabolic resistance in adult specimens [37] were also attempted (see Discussion).

In all cases, the median was employed since the evaluated parameters indicated non-normal distribution (Shapiro-Wilk test, scaled development time: W = 0.91, p-value < 0.01; starvation tolerance: W = 0.82, p-value < 0.01; wing length: W = 0.95, p-value < 0.01; adult female survival: W = 0.97, p-value < 0.01; fecundity: W = 0.93, p-value < 0.01). Scaled development time was described as the percentage of values that remained below and above the Rockefeller counterpart. Starvation tolerance and adult female survival were compared with Kaplan-Meier curves and log-rank tests. Wing length and fecundity were compared with the Kruskal-Wallis test. All the p-values were corrected for multiple comparisons by the Bonferroni method.

Field mosquito populations were contrasted with the reference Rockefeller strain and, since Rockefeller and field populations do not share the same genetic background, fitness parameters were also plotted against the temephos/deltamethrin resistance levels, thus contrasting mosquitoes with a similar genetic background and potentially the same insecticide history and resistance mechanisms. We also investigated global associations between fitness and the frequency of the* kdr* Val1016Ile mutation in the pyrethroid Na_V_ target site. The 16 sampling assays were analysed together with Spearman correlation analyses. All the graphics and analyses were carried out with the statistical software R 3.2.3 [47].

## 3. Results

### 3.1. Mosquito Biology versus Insecticide Resistance Variation

**Figure 1:**
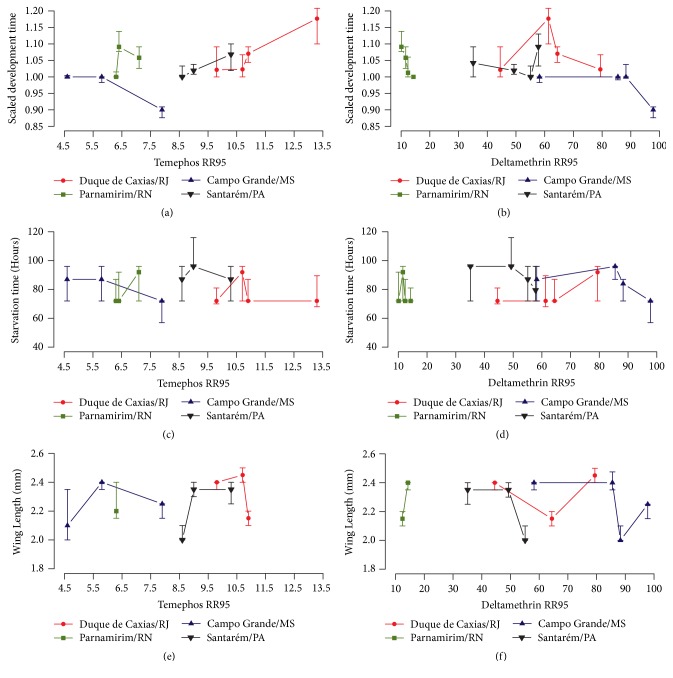
Median scaled development time (a, b), female starvation time (c, d), and female wing length (e, f) versus RR_95_ to temephos and RR_95_ to deltamethrin measured in four Brazilian* Ae. aegypti* populations. Vertical lines correspond to the interquartile range.

**Figure 2:**
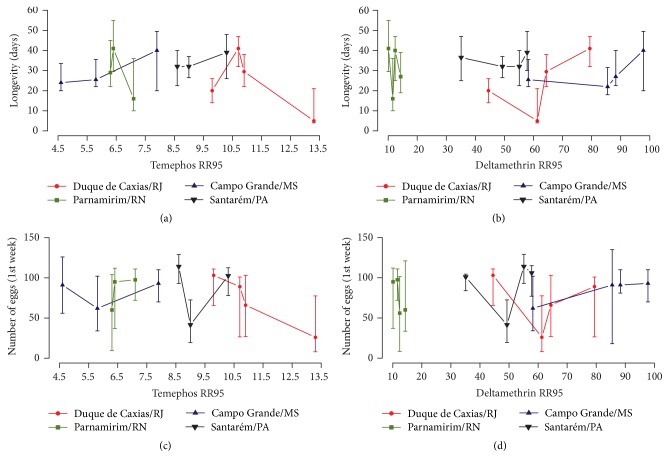
Median adult female survival (a, b) and fecundity for the first week of monitoring (c, d) versus RR_95_ to temephos and RR_95_ to deltamethrin measured in four Brazilian* Ae. aegypti* populations. Vertical lines correspond to the interquartile range.

First, we confronted the parameters of mosquito biology with insecticide resistance levels (i.e., RR_95_). As displayed in Table 2, resistance to temephos presented a consistent dynamic in all mosquito populations analysed, tending to decrease in all localities. Meanwhile, resistance to the adulticide deltamethrin exhibited extremely high levels [33]. Clear associations among the fitness parameters evaluated (scaled development time, starvation tolerance, wing length, adult female survival, and fecundity) and the temephos or deltamethrin resistance ratios (statistics not shown) were not observed (Figures 1 and 2). However, the sample with the highest RR_95_ to temephos (Duque de Caxias Feb/10, RR_95_ of 13.3, Table 2) presented the lowest median values of scaled development time, longevity, and fecundity (Figures 1(a), 2(a) and 2(c)). Duque de Caxias also exhibited the higher metabolic resistance alterations, as evidenced in all ESTs and GST enzymes assays (Table 2). Notwithstanding, no correlations could be identified when data on detoxifying enzymes and fitness were compared. No significant correlations were observed between biological parameters and the* kdr* Val1016Ile Na_V_ allelic frequencies (Spearman correlation analysis, p-value > 0.05) (Supplementary Figure 1).

### 3.2. Comparison among the Different Field Mosquito Populations and Rockefeller

**Figure 3:**
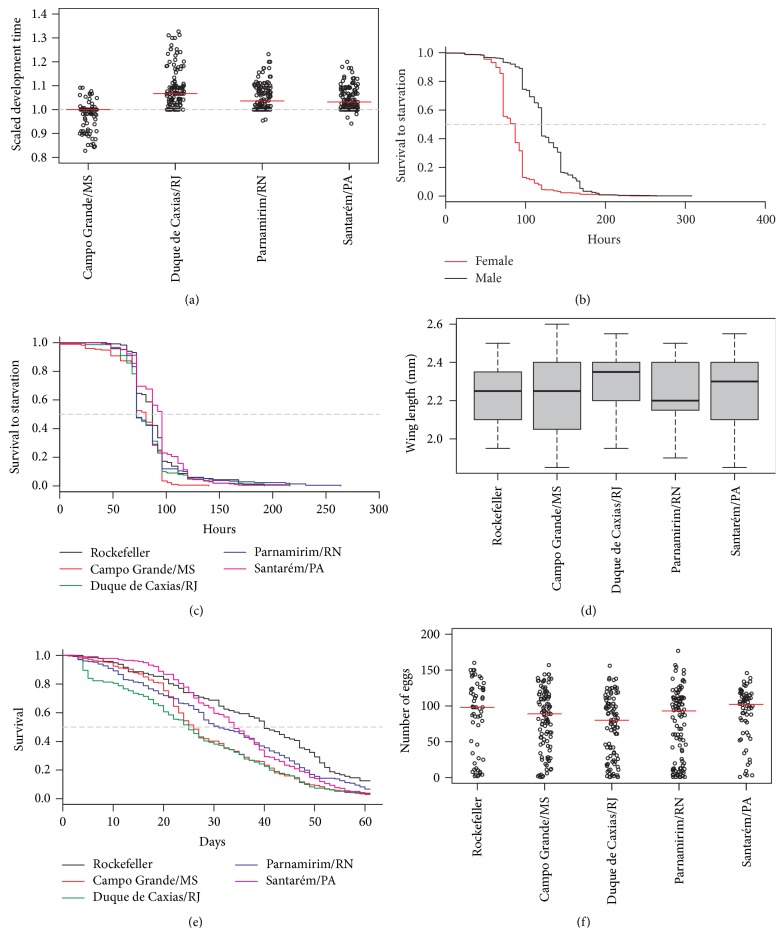
Fitness parameters of four* Ae. aegypti* field populations: scaled development time (a), male and female starvation time (b), female starvation time of each population (c), female wing length (d), female survival (e), and fecundity (f).

Median scaled development time varied from 1 (Campo Grande) to 1.07 (Duque de Caxias), slower than the Rockefeller control. In almost all cases larval development of field populations was slower than that of Rockefeller (Figure 3(a)), except for 40% of the insects from Campo Grande (Figure 3(a)). Regarding starvation tolerance, male mosquitoes survived significantly longer than females in all the 16 samplings (log-rank *χ*^2^ = 724; p-value < 0.01) (Figure 3(b)). The female median starvation tolerance also varied among populations (Figure 3(c)). Median starvation tolerance varied from 72 hours (Duque de Caxias and Parnamirim) to 96 hours (Santarém). Comparing female starvation data of field populations with Rockefeller revealed a lower tolerance for Campo Grande mosquitoes (Log-rank: *χ*^2^ = 14.4, p-value < 0.01) (Figure 3(c)). The median female wing length varied from 2.35 mm (Duque de Caxias) to 2.20 mm (Parnamirim). The median wing size of Duque de Caxias females was considered larger than those of Rockefeller females (Kruskal-Wallis *χ*^2^ = 19.05, p-value < 0.01) (Figure 3(d)).

Median adult female survival varied from 25 (Duque de Caxias) to 34 (Santarém) days, all less than the 40 days observed for Rockefeller. Females from all four field populations survived significantly less than Rockefeller (log-rank versus Santarém: *χ*^2^ = 10.1, p-value < 0.01; log-rank versus Parnamirim: *χ*^2^ = 6.9, p-value < 0.05; log-rank versus Duque de Caxias: *χ*^2^ = 28.4, p-value < 0.01; log-rank versus Campo Grande: *χ*^2^ = 25.2, p-value < 0.01) (Figure 3(e)). Considering the first week of monitoring, in average 60% of Rockefeller mosquitoes laid at least one egg, while this percentage varied between 28.2 and 48.3% in field populations. The median number of eggs per female varied from 80 (Duque de Caxias) to 104 (Santarém). No differences in fecundity were detected among the four field* Ae. aegypti *populations analysed (statistics not shown, Kruskal-Wallis p-value > 0.05) (Figure 3(f)).

## 4. Discussion

In the absence of insecticide applications, resistance alleles can result in energetic costs or fitness disadvantages in comparison with its susceptible counterparts. Attempts to register fitness costs due to insecticide resistance are often based on the selection of insects in the laboratory for increased resistance or on backcrosses with laboratory strains to produce lineages differing only in the resistance traits [11, 28–30, 48, 49]. This approach supposedly enables more accurate measures of changes on the fitness parameters related specifically to insecticide resistance rather than other genetic differences. However, it suffers from an intense inbreeding and the consequent loss of genetic variability which may not reflect insecticide resistance features in the field (reviewed by [50]). In contrast, field populations represent a more realistic situation but exhibit variable genetic backgrounds as the outcome of local adaptation. In this case, differences among susceptible and resistant mosquitoes might be assigned to other characteristics beyond insecticide resistance alleles. Herein, we had the opportunity to correlate resistance and its potentially associated fitness costs on several occasions over the course of one year in four* Ae. aegypti* resistant field populations. By doing so, we were able to estimate whether the seasonal fluctuation of insecticide pressure on each site impacted the fitness cost of insecticide resistance in wild mosquitoes. We also compared field populations with a susceptible laboratory strain (Rockefeller).

The quality of larval environment shapes several traits of adult mosquitoes directly related to vectorial capacity [46, 51]. Additionally, mosquito larvae are more vulnerable to predation, insecticide application, and habitat loss than adults. Survival is another important component of mosquito fitness since higher longevity increases the number of blood meals and subsequently the lifetime number of egg batches [52]. After comparing different samples from the same mosquito population collected over time, no changes in larval development time, survival, and fecundity were found that could be associated with specific insecticide resistance alterations, i.e., fitness reduction when resistance increased and vice versa.

As mentioned above, we evaluated the F1 generation without any artificial insecticide selection in the course of a limited period, one year. This was done to avoid the intense inbreeding and loss of genetic variability typical of highly controlled laboratory assays. We then depended on the natural variation of resistance levels among samples to search for fitness costs oscillations. In the field populations evaluated here, variations of temephos RR_95_ up to 1.26-fold were evident while deltamethrin RR_95_ varied up to 1.59-fold, both values compatible with the expected oscillations of field populations during the period of evaluation. In contrast, controlled laboratory insecticide selection experiments, generally performed for many generations, can produce control/susceptible and resistance strains with very different levels of insecticide resistance [25, 27, 30, 53].

Notwithstanding, field resistant mosquitoes seemed to have a slight fitness disadvantage in comparison to Rockefeller since a development delay was noted in Duque de Caxias and eventually in Parnamirim and Santarém larvae. Additionally, compared to the reference strain, all field-derived adult females exhibited a lower survival. Although laboratory* Ae. aegypti *strains are frequently also adopted as references in resistance fitness costs investigations [25, 26, 48, 49], it is possible that the perceived advantage of Rockefeller mosquitoes over field ones is derived from their laboratory adaptation instead of the absence of an insecticide resistance energy cost. A better comparison would be done with field-derived susceptible mosquitoes, but dissemination of resistance to organophosphate and pyrethroid on Brazilian* Ae. aegypti* populations hampers this approach. Indeed, insecticide resistance reached such high levels that temephos and deltamethrin are no longer recommended for* Ae. aegypti *control in the country [13, 53, 54].

Although it was not possible to assign fitness impairment to any specific insecticide class, due to multiple resistance status and mechanisms in the evaluated populations, Duque de Caxias had the slowest development and lowest survival rates and presented the highest temephos resistance. Resistance of* Ae. aegypti* to organophosphates is usually achieved by the increased production of enzymes such as glutathione-S-transferases, esterases, and mixed function oxidases [53, 55]. The overproduction of these detoxifying enzymes requires that a significant part of insect energy resources be redirected to the machinery related to the metabolism and excretion of the xenobiotic, like the insecticide. This energy trade-off is sustained at the expense of other physiological aspects of the organism, which may reflect negatively on the basic demands of its biology [56]. In particular, this is expected when resistance to organophosphates, such as temephos, and esterases is involved. In insects, esterases have almost no catalytic activity against organophosphates. In this case, esterases act to sequester the insecticide, a phenomenon that requires the production of large amounts of esterase molecules [57]. This situation which has a strong potential impact on vector viability may have occurred with* Ae. aegypti* from Duque de Caxias. Not only do these mosquitoes present the highest RR_95_ to temephos, but also they had the highest average changes in esterases activity, as demonstrated elsewhere [33].

Martins et al. [25], investigating a series of life-trait parameters in five field* Ae. aegypti *populations (F1 generation), verified that when RR_95_ to temephos was higher than 40, a delay in larval development together with a reduction of both adult longevity and fecundity occurred compared to Rockefeller mosquitoes. Diniz et al. [27] also reported a delay in larval development, decreased longevity, and a reduced fecundity in a field population strongly resistant to temephos (RR_95_ > 200). These above described resistant levels are notably higher than those for the populations dealt with herein (temephos RR_95_ 4.6-13.3). However, there are also reports on field populations with lower temephos RR_95_ (7.4 to 19.2) that exhibit decreased blood meal size, number of eggs laid, and rate of inseminated females, when compared to Rockefeller mosquitoes [26].

*Aedes aegypti* resistance to temephos is supposedly acquired essentially through metabolic mechanisms since alterations in acetylcholinesterase, the organophosphate target site, are uncommon in field populations of this species [30, 42, 58]. Since 2009 the Brazilian Ministry of Health stopped the use of temephos as larvicide throughout the country [13, 54]. Theoretically, the absence of insecticide selective pressure would reduce the resistance levels by a rate dependent upon the biological resistance cost [16, 59]. Reversion of organophosphate resistance has already been observed for* Culex pipiens* [60, 61] and* Drosophila melanogaster* [62]. A temephos resistance reduction trend was noted in the four* Ae. aegypti* populations investigated here which is probably the outcome of a lower fitness of resistant insects in the absence of this insecticide [33]. The nonattendance of resistance fitness cost between those mosquito samples in the laboratory may be a consequence of measuring biological traits under optimal conditions, which is a different situation compared to the stressful environment faced by insects in nature [50]. The nutritional status, for example, has been shown to significantly influence the presence and the magnitude of insecticide resistance fitness costs in cockroaches [26].

Regarding pyrethroid resistance, all populations with the exception of Parnamirim exhibited high resistance ratios to deltamethrin (Table 2, [33]). Up to 2009 this was the main adulticide used for* Ae. aegypti* control by Brazilian public managers. Since pyrethroids are freely available in the retail market, they continue to be a major insecticide class for domestic use, although the Ministry of Health gradually started the interruption of deltamethrin use against* Ae. aegypti* in 2009. Overall, deltamethrin resistance did not disclose any temporal trend throughout the one-year sampling period. However, resistance rates varied according to the timing and intensity of dengue outbreaks, corroborating the impact of the domestic chemical control of this urban vector on its resistance status [33]. Changes in the Na_V_ proteins significantly contribute to pyrethroid resistance in several arthropod species [63, 64], including* Ae. aegypti* [65]. Theoretically, mutations in the target sites of neurotoxic insecticides can lead to high levels of resistance which are rapidly selected in the presence of such chemicals [15, 56]. Accordingly,* kdr* mutations in the Na_V_ genes were detected in high frequencies in all populations, except Parnamirim, precisely the population displaying the lowest deltamethrin resistance levels [33]. However, neither of these specific resistance parameters presented a significant connection with the fitness estimates evaluated herein.

The fitness cost of at least one* Ae. aegypti kdr* mutation has already been assessed after introducing the Val1016Ile mutation into the Rockefeller susceptible genetic background. A delay in larval development together with the reduction in both female insemination rate and the number of eggs laid was noted. In addition, the allelic frequency of the Val1016Ile mutation rapidly dropped after 15 generations without any insecticide exposure, further suggesting an associated fitness cost of this mutation [11]. Two out of the four* Ae. aegypti *populations analysed (Duque de Caxias and Campo Grande) presented high Val1016Ile mutation frequencies in all samples (Table 1, [33]). This lack of* kdr* frequency variation over the one-year period is probably related to the absence of detectable fitness differences among field mosquito samples. In opposition, the Val1016Ile mutation was absent, or present at very low levels, in the remaining populations, Santarém and Parnamirim, in the whole period (Table 1, [33]). Accordingly, there were no correlations with biological parameters. Since Santarém and Parnamirim mosquitoes are also resistant to pyrethroids, it is reasonable to assume that other pyrethroid resistance mechanisms are present [33] and may impact the fitness but their effects were not analysed in the scope of this study.

## 5. Conclusions

The comparison of several fitness parameters among samples collected over time from four field* Ae. aegypti* populations did not reveal any correlation between insecticide resistance increase and specific biological losses. However, field resistant mosquitoes exhibit a slight fitness disadvantage when compared with the Rockefeller susceptible strain. This feature might represent a potential insecticide resistance cost. Knowledge about the insecticide resistance evolutionary process in arthropods must be applied in resistance management programs to avoid the loss of effectiveness of the chemical control tools currently available. The occurrence and the magnitude of fitness costs can determine the rate of resistance evolution in field populations as well as the pace to return to a susceptible status after insecticides are removed from the environment [16, 59]. Furthermore, insecticide resistance might entail changes in arthropod biology which can influence the rates of infection, development, and transmission of pathogens harboured by several species of insects [24].

## Figures and Tables

**Table 1:** Cities of mosquito collection as well as climatic, demographic, and epidemiological characteristics.

**Location**	**Region**	**Geographical Coordinates**	**Annual temperature range** ^**a**^ ** (**°**C)**	**Annual precipitation range** ^**a**^ ** (mm)**	**Demographic density (inhabitants/km** ^**2**^ **)** ^**b**^	**Dengue incidence (cases/100,000 inhabitants** ^**c**^ **)**	**Period of mosquito collection**
Campo Grande, Mato Grosso do Sul (MS)	Central-West	20°26'34”S, 54°38'47”W	9.9 to 29.9	34 to 234	97	3,557	Feb, Jun, Oct-2010 and Jan-2011

Duque de Caxias, Rio de Janeiro (RJ)	Southeast	22°47'08”S, 43°18'42”W	17.8 to 29.3	43 a 171	1,828	123.7	Feb, May, Aug and Nov- 2010

Parnamirim, Rio Grande do Norte (RN)	Northeast	5°54'56”S, 35°15'46”W	22 to 29	19 to 212	1,858	55.3	Feb, May, Aug and Dec-2010

Santarém, Pará (PA)	North (Amazon region)	2°26'35”S, 54°42'29”W	23 to 33	33 a 388	12.9	230.7	Apr, Jul, Oct- 2010 and Jan-2011

^a^ Based on daily average temperature during mosquito collection.

^b^ Data obtained from [66–68].

^c^ During each complete mosquito collection period [69].

**Table 2:** Temephos and deltamethrin resistance ratios (RR_95_) and the 95% confidence interval estimated in four Brazilian *Ae. aegypti *populations. The allelic frequencies of the *kdr* mutation Val1016Ile (pyrethroid target site) and enzymatic activities (MFO, EST with three different substrates, and GST) are also shown.

**Municipality/State**	**Sample**	**RR**_**95**_ ** Temephos** ^**a**^	**RR**_**95**_ ** Deltamethrin** ^**a**^	**Na**_**V**_ ** Val1016Ile** ^**a**^	**MFO** ^**a**^	**α** **-EST** ^**a**^	**β** **-EST** ^**a**^	**ρ** **NPA-EST** ^**a**^	**GST** ^**a**^
Campo Grande/MS	Feb-2010	7.9 (7.3-8.6)	97.8 (83.9-119.6)	0.860	3	5	14	14	***76***

	Jun-2010	5.8 (5.3-6.3)	58.2 (54.6-62.7)	0.973	*18*			7	*35*

	Oct-2010	4.6 (4.1-5.3)	88.3 (76.5-106.7)	0.911	3	*27*	*44*	1	*30*

	Jan-2011	4.6 (4.2-5.0)	85.5 (76.8-97.3)	0.817	10	8	*34*	4	*16*

Duque de Caxias/RJ	Feb-2010	13.3 (12.4-14.5)	61.3 (53.1-73.3)	0.929		*38*	*17*	***62***	***81***

	May-2010	10.7 (10.1-11.4)	79.4 (68.7-95.2)	0.923	10			***57***	*33*

	Aug-2010	10.9 (10.2-11.7)	64.4 (54.1-80.1)	0.759	*21*	*45*	*24*	*22*	*46*

	Nov/2010	9.8 (9.2-10.5)	44.5 (40.1-50.0)	0.907	*15*	*24*	7	*26*	*34*

Parnamirim/RN	Feb-2010	7.1 (6.6-7.8)	11.6 (7.9-17.8)	0.017		12	6	*34*	***80***

	May-2010	6.4 (6.1-6.9)	10.1 (8.9-11.8)	0.000	14			*43*	44

	Aug-2010	6.3 (5.9-6.8)	12.4 (11.2-14.1)	0.017	14	*39*	*36*	7	*27*

	Dec-2010	6.3 (6.0-6.7)	14.2 (13.0-16.0)	0.017	5	*31*	13	*23*	***70***

Santarém/PA	Apr-2010	10.2 (8.7-12.2)	57.7 (50.3-67.9)	0.000	7	0	11	11	*48*

	Jul-2010	10.3 (9.7-10.9)	35.1 (32.6-38.3)	0.000	*31*			*20*	*16*

	Oct-2010	8.6 (8-9.2)	55.1 (35.5-82.1)	0.000	2	3	*15*	9	*16*

	Jan-2011	9.0 (8.4-9.6)	49.3 (56.8-44.0)	0.000	14	3	9	3	3

^a^Data originally reported by [33]. Enzymatic activities (MFO, EST with three different substrates, and GST) were classified as normal (regular font), altered (italic and underlined font), or highly altered (italic and bold) if these values ranged, respectively, below 15%, between 15 and 50%, or above 50%.

## Data Availability

The data used to support the findings of this study are available from the corresponding author upon request.

## Abbreviations

RR_95_: Resistance ratio 95
Na_V_: Voltage gated sodium channel
*kdr*: Knockdown resistance
MFO: Mixed function oxidases
EST: Esterases
GST: Glutathione-S-transferases.

## Supplementary Materials

Supplementary Materials Supplementary Text 1. Development time normalization procedure. Each mosquito field sample was normalized by its corresponding Rockefeller specimens to enable comparison among different experimental groups. Supplementary Figure 1. The allelic frequency of the Val1016Ile mutation in the pyrethroid Na_V_ target site alteration versus scaled development time (A), female starvation tolerance (B), female wing length (C), female adult survival (D), and fecundity (E) of* Aedes aegypti* field populations. Red circles and vertical lines correspond to the median and interquartile range, respectively.

## References

[1] Bhatt S., Gething P. W., Brady O. J. (2013). The global distribution and burden of dengue. *Nature*.

[2] Pan American Health Organization: “Number of reported cases of chikungunya fever in the Americas, by country or territory 2016,” http://www2.paho.org/hq/index.php?option=com_topics&amp;view=rdmore&amp;cid=8379&amp;Itemid=40931

[3] Campos G. S., Bandeira A. C., Sardi S. I. (2015). Zika virus outbreak, Bahia, Brazil. *Emerging Infectious Diseases*.

[4] Zanluca C., De Melo V. C. A., Mosimann A. L. P., Dos Santos G. I. V., dos Santos C. N. D., Luz K. (2015). First report of autochthonous transmission of Zika virus in Brazil. *Memórias do Instituto Oswaldo Cruz*.

[5] Secretaria de Vigilância em Saúde - Ministério da Saúde: “Monitoramento dos casos de dengue, febre de chikungunya e febre pelo vírus Zika até a Semana Epidemiológica 52, 2016.” *Bol. Epidemiológico*. vol. 48, no. 3, pp. 1–11, 2017

[6] Oehler E., Watrin L., Larre P. (2014). Zika virus infection complicated by Guillain-Barré syndrome – case report, French Polynesia, December 2013. *Eurosurveillance*.

[7] Calvet G., Aguiar R. S., Melo A. S. (2016). Detection and sequencing of Zika virus from amniotic fluid of fetuses with microcephaly in Brazil: a case study. *The Lancet Infectious Diseases*.

[8] Vega-Rúa A., Zouache K., Girod R., Failloux A.-B., Lourenço-de-Oliveira R. (2014). High level of vector competence of *Aedes aegypti* and aedes albopictus from ten american countries as a crucial factor in the spread of chikungunya virus. *Journal of Virology*.

[9] Fernandes R. S., Campos S. S., Ferreira-de-Brito A. (2016). Culex quinquefasciatus from Rio de Janeiro Is Not Competent to Transmit the Local Zika Virus. *PLOS Neglected Tropical Diseases*.

[10] Focks D. A. A Review of Entomological Sampling Methods and Indicators for Dengue Vectors.

[11] Brito L. P., Linss J. G. B., Lima-Camara T. N. (2013). Assessing the effects of *Aedes aegypti kdr* mutations on pyrethroid resistance and its fitness cost. *PLoS ONE*.

[12] Maciel-de-Freitas R., Avendanho F. C., Santos R. (2014). Undesirable consequences of insecticide resistance following *Aedes aegypti* control activities due to a dengue outbreak. *PLoS ONE*.

[13] Bellinato D. F., Viana-Medeiros P. F., Araújo S. C., Martins A. J., Lima J. B. P., Valle D. (2016). Resistance status to the insecticides temephos, deltamethrin, and diflubenzuron in Brazilian aedes aegypti populations. *BioMed Research International*.

[14] Mallet J. (1989). The evolution of insecticide resistance: Have the insects won?. *Trends in Ecology & Evolution*.

[15] García G. P., Flores A. E., Fernández-Salas I. (2009). Recent rapid rise of a permethrin knock down resistance allele in *Aedes aegypti* in México. *PLOS Neglected Tropical Diseases*.

[16] Crow J. F. (1957). Genetics of Insecticide Resistance to Chemicals. *Annual Review of Entomology*.

[17] Coustau C., Chevillon C., Ffrench-Constant R. (2000). Resistance to xenobiotics and parasites: Can we count the cost?. *Trends in Ecology & Evolution*.

[18] Chevillon C., Raymond M., Guillemaud T., Lenormand T., Pasteur N. (1999). Population genetics of insecticide resistance in the mosquito Culex pipiens. *Biological Journal of the Linnean Society*.

[19] Hoffmann F., Fournier D., Spierer P. (1992). Minigene rescues acetylcholinesterase lethal mutations in Drosophila melanogaster. *Journal of Molecular Biology*.

[20] Grossman M. K., Uc-Puc V., Rodriguez J. (2018). Restoration of pyrethroid susceptibility in a highly resistant. *Biology Letters*.

[21] Carriere Y., Deland J. -., Roff D. A., Vincent C. (1994). Life-history costs associated with the evolution of insecticide resistance. *Proceedings of the Royal Society B Biological Science*.

[22] Berticat C., Boquien G., Raymond M., Chevillon C. (2002). Insecticide resistance genes induce a mating competition cost in Culex pipiens mosquitoes. *Genetics Research*.

[23] Bourguet D., Guillemaud T., Chevillon C., Raymond M. (2004). Fitness costs of insecticide resistance in natural breeding sites of the mosquito Culex pipiens. *Evolution*.

[24] Rivero A., Vézilier J., Weill M., Read A. F., Gandon S. (2010). Insecticide control of vector-borne diseases: When is insecticide resistance a problem?. *PLoS Pathogens*.

[25] Martins A. J., Ribeiro C. D. M., Bellinato D. F., Peixoto A. A., Valle D., Lima J. B. P. (2012). Effect of insecticide resistance on development, longevity and reproduction of field or laboratory selected *Aedes aegypti* populations. *PLoS ONE*.

[26] Belinato T. A., Martins A. J., Valle D. (2012). Fitness evaluation of two Brazilian *Aedes aegypti* field populations with distinct levels of resistance to the organophosphate temephos. *Memórias do Instituto Oswaldo Cruz*.

[27] Diniz F. A. D., Melo-Santos M. A. V., Santos E. M. M. (2015). Fitness cost in field and laboratory *Aedes aegypti* populations associated with resistance to the insecticide temephos. *Parasites & Vectors*.

[28] Berticat C., Bonnet J., Duchon S., Agnew P., Weill M., Corbel V. (2008). Costs and benefits of multiple resistance to insecticides for Culex quinquefasciatus mosquitoes. *BMC Evolutionary Biology*.

[29] Djogbénou L., Noel V., Agnew P. (2010). Costs of insensitive acetylcholinesterase insecticide resistance for the malaria vector Anopheles gambiae homozygous for the G119S mutation. *Malaria Journal*.

[30] Melo-Santos M. A. V., Varjal-Melo J. J. M., Araújo A. P. (2010). Resistance to the organophosphate temephos: Mechanisms, evolution and reversion in an Aedes aegypti laboratory strain from Brazil. *Acta Tropica*.

[31] Leisnham P. T., Sala L. M., Juliano S. A. (2008). Geographic variation in adult survival and reproductive tactics of the mosquito Aedes albopictus. *Journal of Medical Entomology*.

[32] Suman D. S., Tikar S. N., Mendki M. J. (2011). Variations in life tables of geographically isolated strains of the mosquito Culex quinquefasciatus. *Medical and Veterinary Entomology*.

[33] Garcia G. d., David M. R., Martins A. d. (2018). The impact of insecticide applications on the dynamics of resistance: The case of four Aedes aegypti populations from different Brazilian regions. *PLOS Neglected Tropical Diseases*.

[34] Fay R. W., Eliason D. a. (1966). A Preferred Oviposition Site as a Surveillance Method for Aedes aegypti. *Mosq. News*.

[35] Codeço C. T., Lima A. W., Araújo S. C. (2015). Surveillance of Aedes aegypti: Comparison of House Index with Four Alternative Traps. *PLOS Neglected Tropical Diseases*.

[36] Consoli R. A., Oliveira R. L. (1994). *Principais mosquitos de importância sanitária no Brasil*.

[37] Da Silva Soares S., Valle D., Ramos R. P. (2003). resistance of *Aedes aegypti* to organophosphates in several municipalities in the State of Rio de Janeiro and Espírito Santo, Brazil. *The American Journal of Tropical Medicine and Hygiene*.

[38] Kuno G. (2010). Early history of laboratory breeding of *Aedes aegypti* (Diptera: Culicidae) focusing on the origins and use of selected strains. *Journal of Medical Entomology*.

[39] World Health Organization. Division of Vector Biology and Control: “Instructions for determining the susceptibility or resistance of mosquito larvae to insecticides.” *World Health Organization*, Geneva , 1981

[40] WHO - World Health Organization: “Test Procedures for Insecticide Resistance Monitoring in Malaria Vectors, Bio-Efficacy and Persistence of Insecticides on Treated Surfaces,” http://apps.who.int/iris/bitstream/handle/10665/64879/WHO_CDS_CPC_MAL_98.12.pdf?sequence=1, (1998)

[41] Braga I. A., Valle D. (2007). Aedes aegypti: inseticidas, mecanismos de ação e resistência. *Epidemiologia e Serviços de Saúde*.

[42] Montella I. R., Martins A. J., Viana-Medeiros P. F., Lima J. B. P., Braga I. A., Valle D. (2007). Insecticide resistance mechanisms of Brazilian *Aedes aegypti* populations from 2001 to 2004. *The American Journal of Tropical Medicine and Hygiene*.

[43] Martins A. J., Brito L. P., Linss J. G. (2013). Evidence for gene duplication in the voltage-gated sodium channel gene of Aedes aegypti. *Evolution, Medicine, and Public Health*.

[44] Linss J. G. B., Brito L. P., Garcia G. A. (2014). Distribution and dissemination of the Val1016Ile and Phe1534Cys *Kdr* mutations in *Aedes aegypti* Brazilian natural populations. *Parasites & Vectors*.

[45] Agnew P., Hide M., Sidobre C., Michalakis Y. (2002). A minimalist approach to the effects of density-dependent competition on insect life-history traits. *Ecological Entomology*.

[46] Tun-Lin W., Burkot T. R., Kay B. H. (2000). Effects of temperature and larval diet on development rates and survival of the dengue vector Aedes aegypti in north Queensland, Australia. *Medical and Veterinary Entomology*.

[47] R. R Development Core Team: “R: A Language and Environment for Statistical Computing,” http://www.r-project.org, (2011)

[48] Jaramillo-O N., Fonseca-González I., Chaverra-Rodríguez D. (2014). Geometric morphometrics of nine field isolates of Aedes aegypti with different resistance levels to lambda-cyhalothrin and relative fitness of one artificially selected for resistance. *PLoS ONE*.

[49] Alvarez-Gonzalez L. C., Briceño A., Ponce-Garcia G. (2017). Assessing the effect of selection with deltamethrin on biological parameters and detoxifying enzymes in Aedes aegypti (L.). *Pest Management Science*.

[50] Kliot A., Ghanim M. (2012). Fitness costs associated with insecticide resistance. *Pest Management Science*.

[51] Nasci R. S. (1986). The size of emerging and host-seeking Aedes aegypti and the relation of size to blood-feeding success in the field.. *Journal of the American Mosquito Control Association*.

[52] Charlwood J. D. (2003). May the force be with you: measuring mosquito fitness in the field. *Ecological Aspects for Application of Genetically Modified Mosquitoes*.

[53] Moyes C. L., Vontas J., Martins A. J. (2017). Contemporary status of insecticide resistance in the major Aedes vectors of arboviruses infecting humans. *PLOS Neglected Tropical Diseases*.

[54] Chediak M., Pimenta F. G., Coelho G. E. (2016). Spatial and temporal country-wide survey of temephos resistance in Brazilian populations of aedes aegypti. *Memórias do Instituto Oswaldo Cruz*.

[55] Hemingway J., Hawkes N. J., McCarroll L., Ranson H. (2004). The molecular basis of insecticide resistance in mosquitoes. *Insect Biochemistry and Molecular Biology*.

[56] Roush R. T., McKenzie J. A. (1987). Ecological Genetics of Insecticide and Acaricide Resistance. *Annual Review of Entomology*.

[57] Montella I. R., Schama R., Valle D. (2012). The classification of esterases: an important gene family involved in insecticide resistance - A review. *Memórias do Instituto Oswaldo Cruz*.

[58] Marcombe S., Poupardin R., Darriet F. (2009). Exploring the molecular basis of insecticide resistance in the dengue vector *Aedes aegypti*: a case study in Martinique Island (French West Indies). *BMC Genomics*.

[59] Schechtman H., Souza M. O. (2015). Costly inheritance and the persistence of insecticide resistance in Aedes aegypti populations. *PLoS ONE*.

[60] Chevillon C., Bourguet D., Rousset F., Pasteur N., Raymond M. (1997). Pleiotropy of adaptive changes in populations: Comparisons among insecticide resistance genes in Culex pipiens. *Genetics Research*.

[61] Lenormand T., Bourguet D., Guillemaud T., Raymond M. (1999). Tracking the evolution of insecticide resistance in the mosquito Culex pipiens. *Nature*.

[62] Miyo T., Akai S., Oguma Y. (2000). Seasonal fluctuation in susceptibility to insecticides within natural populations of Drosophila melanogaster: Empirical observations of fitness costs of insecticide resistance. *Genes & Genetic Systems*.

[63] Busvine J. R. (1951). Mechanism of resistance to insecticide in Houseflies. *Nature*.

[64] Martinez-Torres D., Chandre F., Williamson M. S. (1998). Molecular characterization of pyrethroid knockdown resistance (kdr) in the major malaria vector *Anopheles gambiae* s.s.. *Insect Molecular Biology*.

[65] Severson D. W., Anthony N. M., Andreev O., ffrench-Constant R. H. (1997). Molecular mapping of insecticide resistance genes in the yellow fever mosquito (Aedes aegypti). *Journal of Heredity*.

[66] Prefeitura Municipal de Santarém: “Informações municipais de Santarém, SEMMA_CIAM,” http://www.santarem.pa.gov.br/arquivosdb/basico1/0.668764001357580532__informacoes_2.pdf

[67] Portal da Prefeitura de Parnamirim: “Portal de transparência, Estatísticas e mapas,” http://www.parnamirim.rn.gov.br/mapas.jsp

[68] Instituto Brasileiro de Geografia e Estatística - IBGE: “Cidades,” http://cidades.ibge.gov.br/xtras/home.php?lang=

[69] Ministério da Saúde: “Sistema de Informação de Agravos de Notificação (SINAN),” http://dtr2004.saude.gov.br/sinanweb/

