# Supplementary material for: Insecticide Resistance and Fitness: The Case of Four Aedes aegypti Populations from Different Brazilian Regions
Source: Biomed Res Int. 2018 Oct 9;2018:6257860. doi: 10.1155/2018/6257860 (PMC6198578; doi:10.1155/2018/6257860)

**Supplementary text 1. Development time normalization procedure.** Each mosquito field sample was normalized by its corresponding Rockefeller specimens to enable comparison among different experimental groups.

Due to the great variation in immature development assays, including those with the Rockefeller larvae, each evaluated sampling was normalized as follows:

(a) the experimental groups, consisting of only the specimens that survived to the adult stage (out of the 120 tested), were ranked in ascending order of development time, the same was done with the corresponding Rockefeller insects (from an initial sample of 36);

| Development time of field mosquitoes (hours)* | Development time of corresponding Rockefeller mosquitoes (hours)* |
| --- | --- |
| 216 | 216 |
| 221 | 220 |
| 236 | 225 |
| 240 | 240 |
| 245 |  |
| 249 |  |
| 260 |  |
| 308 |  |
| 388 |  |
| 404 |  |

* Illustrative example, not real data.

(b) data from the experimental group were divided into N subgroups respecting the increasing order, N the number of Rockefeller larvae that completed development, each subgroup differing by at most one specimen; example:

N from field population = 10

N from Rockefeller = 4

10 ÷ 4 = 2 and there were 2 left over. So, we will have 2 groups with 2 specimens and 2 groups with 3 specimens (indicated by different colors in the table below). The left overs were always distributed in the last groups.

| Development time of field mosquitoes (hours)* | Development time of corresponding Rockefeller mosquitoes (hours)* |
| --- | --- |
| 192 | 216 |
| 198 | 220 |
| 236 | 225 |
| 240 | 240 |
| 245 |  |
| 249 |  |
| 260 |  |
| 308 |  |
| 388 |  |
| 404 |  |

(c) the mean development time of each experimental subgroup was divided by the corresponding Rockefeller value, considering the ranking order (indicated by different colors in the table below);

| Development time of field mosquitoes (hours)* | Mean of subgroups | Development time of corresponding Rockefeller mosquitoes (hours)* | Scaled development time |
| --- | --- | --- | --- |
| 192 | 195 | 216 | 0.90 |
| 198 |  |  |  |
| 236 | 238 | 220 | 1.08 |
| 240 |  |  |  |
| 245 | 251.3 |  |  |
| 249 |  | 225 | 1.11 |
| 260 |  |  |  |
| 308 | 366.6 |  |  |
| 388 |  | 240 | 1.53 |
| 404 |  |  |  |

Thus, the values generated for the experimental groups, called 'scaled time values', are a measure of how much each field population development time deviates from its corresponding Rockefeller referential. Values above and below 1.0 indicate, respectively, slower or faster development than the Rockefeller strain.

**Supplementary figure 1. The allelic frequency of the Val1016Ile mutation in the pyrethroid Na_V_ target site alteration versus scaled development time (A), female starvation tolerance (B), female wing length (C), female adult survival (D) and fecundity (E) of *Aedes aegypti* field populations.** Red circles and vertical lines correspond to the median and interquartile range, respectively.


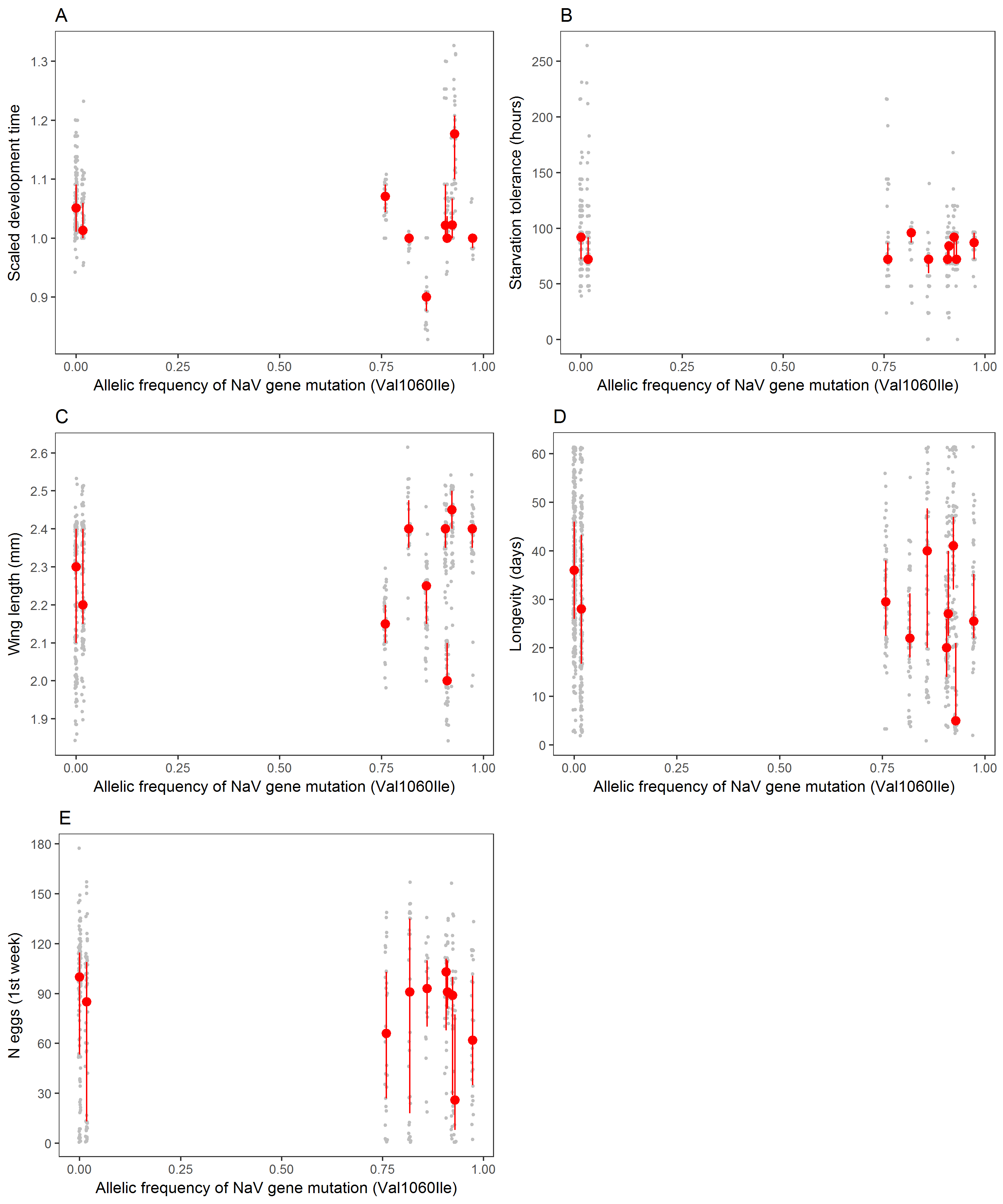

Supplement: Supplementary Materials — Supplementary Text 1. Development time normalization procedure. Each mosquito field sample was normalized by its corresponding Rockefeller specimens to enable comparison among different experimental groups. Supplementary Figure 1. The allelic frequency of the Val1016Ile mutation in the pyrethroid NaV target site alteration versus scaled development time (A), female starvation tolerance (B), female wing length (C), female adult survival (D), and fecundity (E) of Aedes aegypti field populations. Red circles and vertical lines correspond to the median and interquartile range, respectively. [file 6257860.f1.doc]
